# Associations between metabolic dysfunction-associated fatty liver disease, chronic kidney disease, and abdominal obesity: a national retrospective cohort study

**DOI:** 10.1038/s41598-024-63386-0

**Published:** 2024-06-02

**Authors:** Chao Cen, Zhongwen Fan, Xinjiang Ding, Xinyue Tu, Yuanxing Liu

**Affiliations:** Department of Hepatobiliary and Pancreatic Surgery, The First Affiliated Hospital, Zhejiang University School of Medicine, Hangzhou, 310003 China

**Keywords:** Abdominal obesity, Metabolic dysfunction-associated fatty liver disease (MAFLD), Chronic kidney disease (CKD), Diabetes mellitus, Liver fibrosis, Mortality, NHANES III, Chronic kidney disease, Non-alcoholic fatty liver disease, Obesity, Risk factors

## Abstract

Metabolic dysfunction-associated fatty liver disease (MAFLD) and chronic kidney disease (CKD) present notable health challenges, however, abdominal obesity has received scant attention despite its potential role in exacerbating these conditions. Thus, we conducted a retrospective cohort study using the National Health and Nutrition Examination Surveys III (NHANES III) of the United States from 1988 to 1994 including 9161 participants, and mortality follow-up survey in 2019. Statistical analyze including univariable and multivariable Logistic and Cox regression models, and Mediation effect analyze were applied in study after adjustment for covariates. Our findings revealed that individuals with both abdominal obesity and MAFLD were more likely to be female, older and exhibit higher prevalence of advanced liver fibrosis (7.421% vs. 2.363%, *p* < 0.001), type 2 diabetes mellitus (T2DM) (21.484% vs. 8.318%, *p* < 0.001) and CKD(30.306% vs. 16.068%, *p* < 0.001) compared to those with MAFLD alone. MAFLD (adjusted OR: 1.392, 95% CI 1.013–1.913, *p* = 0.041), abdominal obesity (adjusted OR 1.456, 95% CI 1.127–1.880, *p* = 0.004), abdominal obesity with MAFLD (adjusted OR 1.839, 95% CI 1.377–2.456, *p* < 0.001), advanced fibrosis(adjusted OR 1.756, 95% CI 1.178–2.619, *p* = 0.006) and T2DM (adjusted OR 2.365, 95% CI 1.758–3.183, *p* < 0.001) were independent risk factors of CKD. The abdominal obese MAFLD group had the highest all-cause mortality as well as mortality categorized by disease during the 30-year follow-up period. Indices for measuring abdominal obesity, such as waist circumference (WC), waist-hip ratio (WHR), and lipid accumulation product (LAP), elucidated a greater mediation effect of MAFLD on CKD compared to BMI on CKD (proportion mediation 65.23%,70.68%, 71.98%, respectively vs. 32.63%). In conclusion, the coexistence of abdominal obesity and MAFLD increases the prevalence and mortality of CKD, and abdominal obesity serves as a mediator in the association between MAFLD and CKD.

## Introduction

Non-alcoholic fatty liver disease (NAFLD) is one of the most common chronic liver diseases, affecting nearly 30% of the global adult population and having a significant impact on global health and the economy^1^. In 2020, there was a proposal to rename and redefine NAFLD as metabolic-associated fatty liver disease (MAFLD) in order to better characterize the underlying pathophysiology and associated metabolic abnormalities. The proposed criteria for diagnosing MAFLD include evidence of hepatic steatosis along with one of the following three criteria: overweight or obesity, presence of type 2 diabetes mellitus (T2DM), or evidence of metabolic dysregulation^2^. This condition has been recognized as a significant global health concern due to its increasing prevalence and potential to progress to more severe liver diseases, including advanced liver fibrosis and liver cancer^3^.

Chronic kidney disease (CKD) is another prevalent and progressive condition that affects millions of individuals worldwide^4^. It is characterized by a gradual loss of kidney function, leading to various complications and an increased risk of cardiovascular events and mortality^5^. The etiology of CKD is multifactorial, involving both non-modifiable factors such as age and genetic predisposition, as well as modifiable factors such as diabetes mellitus, hypertension, and obesity, which are also the metabolic risk factors shared with MAFLD^6^. Several studies have demonstrated that individuals with MAFLD are more likely to have and develop CKD compared with those without MAFLD^7,8^.

Abdominal obesity, typified by the accumulation of visceral fat, has been found to have a stronger correlation with metabolic abnormalities and is considered a more reliable predictor of metabolic and cardiovascular diseases compared to overall obesity, which may also encompass MAFLD and CKD^9^. Previous studies have substantiated a strong association between obesity and both MAFLD and CKD^10,11^. However, these studies have predominantly focused on general obesity measures such as body mass index (BMI), which do not offer a comprehensive assessment of abdominal fat distribution. Waist circumference(WC) is commonly used to measure abdominal obesity, and meanwhile newer indices such as body shape index (ABSI), body roundness index (BRI), visceral fat index (VAI), and lipid accumulation product (LAP) have emerged in recent years to measure abdominal obesity or visceral fat^12^. Although previous research has extensively examined the independent links between MAFLD and CKD with obesity, the potential mediating role of abdominal obesity between MAFLD and CKD remains underexplored. Understanding this potential association is crucial in identifying effective interventions and developing targeted strategies to prevent and manage both MAFLD and CKD.

Therefore, the objective of this national retrospective cohort study is to investigate the relationship between MAFLD, CKD and abdominal obesity, as well as to analyze mortality outcomes stratified by cause of death during a 30-year follow-up period. We extracted pertinent associations from extensive population data to enhance our understanding of the intricate mechanisms linking MAFLD, abdominal obesity, and CKD, shed light on the potential mediation effects of abdominal obesity of these conditions, and ultimately provide valuable insights for clinical practice and public health interventions.

## Materials and methods

### Study population

The National Health and Nutrition Examination Survey (NHANES) is a national survey managed by the National Center for Health Statistics (NCHS) at the U.S. Centers for Disease Control and Prevention (CDC). Samples in NHANES represented the health and nutritional status of the general U.S. population well and employed a carefully conducted multistage and stratification probability design. NHANES is designed to monitor health and nutritional status in the US through the collection of demographic, dietary, physical examination, hepatic ultrasound, laboratory and questionnaire data from adults and children. All participants gave written informed consent. NHANES is publicly available at www.cdc.gov/nchs/nhanes/.

For this study, we utilized the Third National Health and Nutrition Examination Survey (NHANES III) and the Third National Health and Nutrition Examination Survey Mortality Follow-up studies. The survey was conducted from 1988 to 1994, and the mortality follow-up study was a prospective study of the vital status of all participants aged 20 and older to December 2019. The Participants’ length of survival was determined by the amount of time between the date of completion of the NHANESIII survey to time of death or 31 December 2019, whichever came first. All-cause mortality was defined as any reason for death. Data on mortality due to specific causes was collected either, such as cardiovascular and cerebrovascular diseases (CVD), diabetes mellitus and kidney diseases (include nephritis, nephrotic syndrome and nephrosis) related mortality.

General demographic and socioeconomic characteristics such as age, sex, race and ethnicity (Non-Hispanic White, Non-Hispanic Black, Mexican American, Other), marital status (single, married, divorced/separated, other/widowed), military service, ratio of family income to poverty (PIR), and sedentary behavior, smoke and alcohol use were obtained. All these variables were self-reported as per the design of NHANES. Physical and blood measurements including BMI, WC, VAI, LAP, white blood cell (WBC), red blood cell (RBC), hemoglobin(HB), platelet (PLT), Na^+^, K^+^, Cl^−^, Ca^2+^, blood urea nitrogen (BUN), total bilirubin (TB), serum creatinine (Scr), γ-glutamyl transpeptidase (GGT) , lactate dehydrogenase (LDH), aspartate aminotransferase (AST), alanine aminotransferase (ALT), alkaline phosphatase (ALP), albumin, globulin, total protein (TP), total cholesterol(Tc), high-density lipoprotein cholesterol (HDL-C), low-density lipoprotein cholesterol (LDL-C), triglycerides(TG), estimated glomerular filtration rate (eGFR), serum glucose, homeostatic model assessment of insulin resistance (HOMA-IR), urinary albumin, albumin-to-creatinine ratio (ACR) were obtained from the laboratory tests.

### Inclusion/exclusion criteria

**Figure 1:**
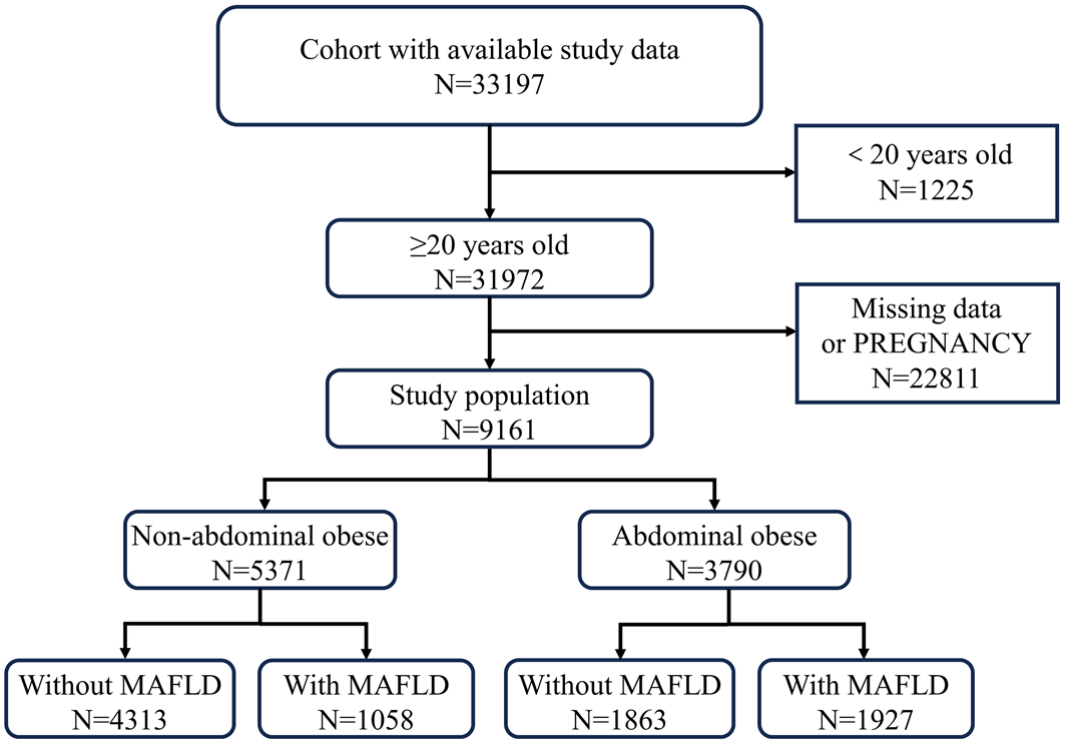
Flowchart.

33197 participants in total subjects from the NHANES III were included. Among them, we excluded subjects who were aged below 20 years old (N = 1225), pregnant(as pregnancy can significantly impact waist circumference and other body measurements, leading to potential confounding factors in our study) or lacked laboratory and ultrasound data(N = 22811), after which a total of 9161 participants remained. Finally, we categorized participants based on the presence or absence of MAFLD and abdominal obesity (Fig. 1).

### Definition of MAFLD, abdominal obesity and CKD

The established 2020 criteria for MAFLD include evidence of hepatic steatosis plus one of the following: overweight (BMI ≥ 25 and < 30 kg/m^2^)/obesity (BMI ≥ 30 kg/m^2^), T2DM, or t at least two metabolic risk abnormalities. Metabolic risk abnormalities consisted of: (1) waist circumference ≥ 102 cm in male and ≥ 88 cm female, (2) blood pressure ≥ 130/85 mmHg or specific drug treatment, (3) plasma triglycerides ≥ 150 mg/dl (≥ 1.70 mmol/L) or specific drug treatment, (4) plasma HDL-cholesterol < 40 mg/dl (< 1.0 mmol/L) for male and < 50 mg/dl (< 1.3 mmol/L) for female or specific drug treatment, (5) prediabetes (fasting glucose levels 100–125 mg/dl [5.6 to 6.9 mmol/L] or hemoglobin A1c [HbA1c[ 5.7–6.4% [39 to 47 mmol/mol]), (6) homeostasis model assessment of insulin resistance (HOMA-IR) score ≥ 2.5, (7) and/or plasma high-sensitivity C-reactive protein level > 2 mg/L. T2DM in this study was defined as a history of diabetes mellitus or HbA1c ≥ 6.5% or serum glucose level ≥ 7.0mmol/L^13^. Hypertension was defined as a history of hypertension or blood pressure ≥ 140/90 mmHg^2^.

Hepatic steatosis was determined in NHANES III participants using the Hepatic Steatosis Ultrasound Examination (HSUE). The ultrasonographic assessments were reported as normal, mild, moderate, or severe hepatic steatosis. All ultrasound personnel received training in the standardized procedures, and they were supervised periodically. Abiding by quality control procedures, reliability results (intra-rater and inter-rater) were calculated. The intra-rater reliability was found to be 91.3% (kappa 0.77) and the inter-rater reliability was found to be 88.7% (kappa 0.70)^14^ .

The advanced fibrosis was defined as Fibrosis-4 index (FIB-4) ≥ 2.67, NAFLD fibrosis score (NFS) ≥ 0.676 and AST to platelet ratio index (APRI) ≥ 0.918^15,16^. FIB-4 = Age[years] × AST[U/L])/(platelet [10^9^] × ALT[U/L]); NFS =  − 1.675 + (0.037 × Age[years]) + (0.094 × BMI) + (1.13 × IFG/diabetes [yes = 1, no = 0]) + (0.99 × AST/ALT) − (0.013 × platelet [10^9^/l]) − (0.66 × albumin[g/dL]); APRI = ([AST/upper limit of normal]/platelet count [10^9^/l]) × 100^17^.

Abdominal obesity is determined according to the WC thresholds. The cut-off points for abdominal obesity for men and women were ≥ 102 cm and ≥ 88 cm, respectively^18^. In addition to BMI and WC, there are many other obesity measurement indexes, including traditional indexes such as waist-hip ratio (WHR) and waist-to-height ratio (WHtR), as well as new indexes such as a ABSI, BRI, VAI and LAP. WHR = WC (cm)/ Hip Circumference (cm), WHtR = WC (cm)/Height (cm). ABSI = WC (m)/[BMI (kg/m^2^)^2/3^ × Height (m)^1/2^]; BRI = 364.2 − 365.5 × [1 − (WC(m)/2π)^2^/(0.5 × Height (m)^2^]^1/2^^19^. VAI is the integration of BMI, WC, TG and HDL: for male, VAI = [WC (cm)/[39.68 + 1.88 × BMI (kg/m^2^)]] × TG (mmol/L)/1.03 × [1.31/HDL − C (mmol/L)], for female, VAI = [WC (cm)/[36.58 + 1.89 × BMI (kg/m^2^)]] × TG (mmol/L)/ 0.81 × [1.52/HDL − C (mmol/L)]^20^. LAP is the indicator used to evaluate lipid accumulation, and it combined WC and triglycerides (TGs): for males, LAP = (WC[cm] − 65) × TG[mmol/L]; for females, LAP = (WC [cm] − 58) × TG[mmol/L]^21^.

CKD was defined as an estimated glomerular filtration rate (eGFR) < 60 mL/min/1.73 m2 (CKD-EPI) and/or ACR ≥ 30 mg/g^22,23^.

### Statistical analyses

We followed CDC guidelines rigorously during all statistical analyses and used a suitable sample weight for each participant to account for the NHANES complex multistage cluster survey design^24^.

The included subjects were divided into four groups: the non- abdominal obese and non-MAFLD group, non-abdominal obese and MAFLD group, the abdominal obese and non-MAFLD group, the abdominal obese and MAFLD group. Characteristics of participants by the presence of abdominal obese and MAFLD group were reported as weighted percentages(95%CI) or mean with standard error (SE), aimed to make the sample better reflect the characteristics of the population. The continuous variables were compared using Student’s t-test, and categorical variables using χ2 test. Multivariable logistics regression analysis was used to assess independent risk factors. All tests were two-tailed and results with a *p* < 0.05 were considered statistically significant. All calculations were conducted by SPSS version 25.0 (IBM Corp., Armonk, NY, USA) and STATA version 18.0 (Stata Corp., College Station, TX, USA).

We calculated the prevalence of our divided four groups among the overall cohort of patients in relevant subgroups such as by T2DM, CKD, and advanced fibrosis by NFS, we also calculated the prevalence of CKD groups in subgroups as well. We further performed univariable and adjusted multivariable logistic regression models to determine risks factors associated with CKD. We reported the univariate and multivariate odd ratios (OR) as 95% confidence intervals (CI).

Next, we estimated overall mortality rates in study population and in subgroups using the Kaplan–Meier methods. Cox regression analysis was used to compare mortality of different groups and Cox regression models were used to test hazard ratio (HR) of known risk factors for kidney disease-related mortality after adjustment for potential confounders (sex, race, age, marital status, military service, sedentary behavior, weight category by BMI, advanced fibrosis by NFS, proteinuria, LDL, TRI, Tc, albumin).

Mediation analysis is a statistical method used to assess the underlying mechanisms through which an independent variable affects a dependent variable. We used mediation analysis to explore whether the associations of MAFLD with CKD were mediated by abdominal obesity (mediator: BMI, WC, WHR, WHtR, ABSI, BRI, LAP, and VAP). The direct effect (DE) represented the effects of MAFLD on CKD without a mediator. The indirect effect (IE) represented the effects of MAFLD on CKD through the mediator. A significant IE is indicative of a mediation effect. The proportion of mediation was calculated by using IE divided by TE (total effect).

## Results

### Baseline characteristics of subjects

Baseline characteristics are shown in Table 1. Among the 9161 participants, 48.030% were men, the mean age was 43.227 ± 0.164 years old. Participants with MAFLD were found to be older (*p* < 0.001) and had a higher incidence of CKD (*p* < 0.001) compared to those without MAFLD. Several significant differences were observed in other demographic factors among the four groups, such as sex, age, race, PIR, marital status, and military service (*p* < 0.001). When categorizing MAFLD patients based on abdominal obesity presence, the abdominal obese MAFLD group tended to be female, older, exhibiting sedentary behavior, and having higher values of BMI, LAP, VAP, Tc, TRI, LDL, et al. compared to the non-abdominal obese MAFLD group (*p* < 0.001). As expected, the abdominal obese MAFLD group exhibited a higher prevalence of T2DM, advanced liver fibrosis (by NFS and APRI), proteinuria and CKD than other three groups (*p* < 0.001).

**Table 1:** Demographic, clinical, and laboratory characteristics of participants divided by abdominal obesity and MAFLD.

Characteristics	Total	Non-abdominal obesity		Abdominal obesity		*P*-Value
		without MAFLD	with MAFLD	without MAFLD	with MAFLD	
		Group1	Group2	Group3	Group4	
	N = 9161	N = 4313	N = 1058	N = 1863	N = 1927	
Sex						< 0.001
Male	48.030(47.007–49.054)	57.060(55.577–58.531)	69.282(66.434–71.990)	24.047(22.160–26.041)	39.336(37.177–41.537)	
Female	51.970(50.946–52.993)	42.940(41.469–44.423)	30.718(28.010–33.566)	75.953(73.959–77.840)	60.664(58.463–62.823)	
Age						< 0.001
< 65	86.355(85.637–87.043)	90.633(89.726–91.467)	87.240(85.091–89.119)	82.179(80.375–83.851)	80.332(78.497–82.047)	
≥ 65	13.645(12.957–14.363)	9.367(8.533–10.274)	12.760(10.881–14.909)	17.821(16.149–19.625)	19.668(17.953–21.503)	
Race						< 0.001
Non-Hispanic White	37.965(36.977–38.964)	40.111(38.658–41.583)	31.096(28.378–33.952)	37.037(34.872–39.256)	37.831(35.691–40.019)	
Non-Hispanic Black	28.469(27.553–29.402)	28.634(27.304–30.002)	24.669(22.164–27.358)	36.286(34.131–38.496)	22.626(20.812–24.549)	
Mexican American	29.451(28.526–30.393)	26.617(25.319–27.957)	39.698(36.79–42.68)	23.564(21.691–25.546)	35.859(33.747–38.027)	
Other race	4.115(3.727–4.542)	4.637(4.048–5.307)	4.537(3.435–5.970)	3.113(2.414–4.006)	3.684(2.930–4.624)	
PIR						< 0.001
Blank but applicable	8.733(8.172–9.328)	8.161(7.381–9.017)	9.735(8.089–11.674)	9.232(7.999–10.634)	8.978(7.780–10.339)	
< 1.0	20.238(19.428–21.073)	17.389(16.287–18.550)	23.062(20.622–25.698)	21.846(20.028–23.781)	23.508(21.668–25.454)	
≥ 1.0	71.029(70.092–71.95)	74.449(73.126–75.729)	67.202(64.313–69.967)	68.921(66.781–70.983)	67.514(65.389–69.570)	
Marital status						< 0.001
Legally married	60.059(59.052–61.058)	56.504(55.019–57.977)	63.989(61.048–66.828)	61.138(58.903–63.327)	64.816(62.655–66.918)	
Divorced/separated	12.149(11.496–12.834)	10.758(9.868–11.719)	11.909(10.091–14.004)	14.654(13.119–16.334)	12.974(11.545–14.549)	
Never married	17.935(17.162–18.734)	24.67(23.406–25.979)	16.635(14.51–19.002)	12.185(10.775–13.75)	9.133(7.926–10.504)	
Other(a)	9.857(9.263–10.485)	8.069(7.292–8.920)	7.467(6.029–9.214)	12.024(10.623–13.581)	13.077(11.644–14.658)	
Military service						< 0.001
Blank but applicable	0.546(0.414–0.719)	0.533(0.355–0.801)	0.567(0.255–1.257)	0.537(0.289–0.995)	0.571(0.316–1.028)	
No	15.642(14.913–16.401)	15.419(14.371–16.527)	21.55(19.175–24.131)	11.648(10.268–13.186)	16.762(15.159–18.497)	
Yes	83.812(83.043–84.552)	84.048(82.925–85.111)	77.883(75.281–80.283)	87.815(86.25–89.225)	82.667(80.911–84.293)	
Sedentary behavior						< 0.001
No	71.608(70.675–72.522)	77.556(76.286–78.777)	70.794(67.980–73.457)	66.130(63.948–68.245)	64.037(61.868–66.151)	
Yes	28.392(27.478–29.325)	22.444(21.223–23.714)	29.206(26.543–32.020)	33.870(31.755–36.052)	35.963(33.849–38.132)	
Weight category by BMI						< 0.001
Non-obese	74.097(73.189–74.984)	97.728(97.238–98.133)	95.274(93.818–96.400)	49.705(47.436–51.975)	33.160(31.093–35.295)	
Obese	25.903(25.016–26.811)	2.272(1.867–2.762)	4.726(3.600–6.182)	50.295(48.025–52.564)	66.840(64.705–68.907)	
Advanced fibrosis by FIB-4						0.129
No	99.465(99.293–99.596)	99.629(99.395–99.773)	99.338(98.619–99.684)	99.463(99.005–99.711)	99.170(98.649–99.491)	
Yes	0.535(0.404–0.707)	0.371(0.227–0.605)	0.662(0.316–1.381)	0.537(0.289–0.995)	0.830(0.509–1.351)	
Advanced fibrosis by NFS						< 0.001
No	96.420(96.019–96.781)	98.609(98.212–98.918)	97.637(96.526–98.399)	94.632(93.512–95.569)	92.579(91.320–93.668)	
Yes	3.580(3.219–3.981)	1.391(1.082–1.788)	2.363(1.601–3.474)	5.368(4.431–6.488)	7.421(6.332–8.680)	
Advanced fibrosis by APRI						< 0.001
No	98.843(98.602–99.043)	99.258(98.953–99.475)	97.732(96.638–98.475)	99.249(98.735–99.554)	98.132(97.421–98.650)	
Yes	1.157(0.957–1.398)	0.742(0.525–1.047)	2.268(1.525–3.362)	0.751(0.446–1.265)	1.868(1.350–2.579)	
T2DM						< 0.001
No	91.169(90.570–91.733)	97.079(96.532–97.541)	91.682(89.860–93.202)	90.284(88.854–91.549)	78.516(76.625–80.293)	
Yes	8.831(8.267–9.430)	2.921(2.459–3.468)	8.318(6.798–10.140)	9.716(8.451–11.146)	21.484(19.707–23.375)	
IFG						< 0.001
No	94.400(93.910–94.853)	97.055(96.507–97.520)	92.344(90.580–93.800)	94.096(92.930–95.079)	89.881(88.452–91.150)	
Yes	5.600(5.147–6.090)	2.945(2.480–3.493)	7.656(6.200–9.420)	5.904(4.921–7.070)	10.119(8.850–11.548)	
Proteinuria						< 0.001
Normal range	90.372(89.751–90.960)	94.134(93.392–94.797)	91.304(89.449–92.859)	87.332(85.743–88.767)	84.38(82.689–85.933)	
Microalbuminuria	8.012(7.474–8.586)	5.078(4.461–5.774)	7.561(6.114–9.317)	10.199(8.904–11.657)	12.714(11.299–14.277)	
Macroalbuminuria	1.616(1.377–1.895)	0.788(0.564–1.101)	1.134(0.645–1.987)	2.469(1.854–3.281)	2.906(2.243–3.758)	
CKD						< 0.001
No	80.559(79.736–81.357)	87.572(86.554–88.524)	83.932(81.594–86.024)	73.645(71.596–75.596)	69.694(67.603–71.706)	
Yes	19.441(18.643–20.264)	12.428(11.476–13.446)	16.068(13.976–18.406)	26.355(24.404–28.404)	30.306(28.294–32.397)	
Age	43.227 ± 0.164	38.953 ± 0.230	42.121 ± 0.471	46.797 ± 0.359	49.948 ± 0.329	< 0.001
BMI(kg/m2)	27.208 ± 0.060	23.658 ± 0.046	24.953 ± 0.102	30.82 ± 0.111	32.898 ± 0.132	< 0.001
WC(cm)	92.891 ± 0.151	83.324 ± 0.137	87.933 ± 0.289	102.067 ± 0.230	108.153 ± 0.271	< 0.001
VAI	2.315 ± 0.026	1.555 ± 0.024	2.511 ± 0.075	2.532 ± 0.051	3.697 ± 0.078	< 0.001
LAP	54.754 ± 0.605	28.356 ± 0.386	48.218 ± 1.304	69.117 ± 1.164	103.541 ± 1.891	< 0.001
WHR	0.915 ± 0.001	0.877 ± 0.001	0.918 ± 0.002	0.938 ± 0.002	0.976 ± 0.002	< 0.001
WHtR	0.557 ± 0.001	0.495 ± 0.001	0.522 ± 0.002	0.619 ± 0.001	0.654 ± 0.002	< 0.001
ABSI	0.0798 ± 0.0001	0.0781 ± 0.0001	0.0796 ± 0.0001	0.0813 ± 0.0001	0.0823 ± 0.0001	< 0.001
BRI	4.644 ± 0.021	3.32 ± 0.014	3.841 ± 0.030	5.943 ± 0.034	6.794 ± 0.041	< 0.001
WBC	7.143 ± 0.024	6.9 ± 0.032	6.975 ± 0.062	7.37 ± 0.064	7.557 ± 0.048	< 0.001
RBC	4.669 ± 0.005	4.674 ± 0.007	4.752 ± 0.015	4.576 ± 0.01	4.702 ± 0.011	< 0.001
HB(g/dL)	13.99 ± 0.016	14.107 ± 0.022	14.294 ± 0.048	13.522 ± 0.033	14.011 ± 0.034	< 0.001
PLT	278.771 ± 0.733	272.104 ± 0.992	271.613 ± 2.139	291.172 ± 1.75	285.635 ± 1.675	< 0.001
Tc(mmol/L)	5.268 ± 0.012	5.037 ± 0.016	5.22 ± 0.036	5.521 ± 0.026	5.567 ± 0.026	< 0.001
TG(mmol/L)	1.578 ± 0.013	1.239 ± 0.012	1.807 ± 0.042	1.606 ± 0.024	2.186 ± 0.039	< 0.001
LDL(mmol/L)	1.446 ± 0.018	1.405 ± 0.026	1.313 ± 0.052	1.588 ± 0.043	1.471 ± 0.041	< 0.001
HDL(mmol/L)	1.314 ± 0.004	1.392 ± 0.006	1.246 ± 0.013	1.318 ± 0.009	1.174 ± 0.008	< 0.001
Na(mmol/L)	141.363 ± 0.025	141.414 ± 0.035	141.456 ± 0.071	141.248 ± 0.06	141.312 ± 0.056	0.023
K(mmol/L)	4.023 ± 0.003	4.029 ± 0.005	4.038 ± 0.01	4.016 ± 0.007	4.007 ± 0.008	0.140
Cl(mmol/L)	104.67 ± 0.034	104.71 ± 0.047	104.53 ± 0.101	104.871 ± 0.078	104.463 ± 0.079	< 0.001
Ca(mmol/L)	2.32 ± 0.001	2.326 ± 0.002	2.324 ± 0.003	2.311 ± 0.003	2.316 ± 0.002	< 0.001
BUN(mmol/L)	4.934 ± 0.019	4.873 ± 0.026	4.897 ± 0.050	4.945 ± 0.045	5.08 ± 0.044	0.001
TB(μmol/L)	10.158 ± 0.060	10.74 ± 0.090	10.942 ± 0.208	8.753 ± 0.103	9.786 ± 0.132	< 0.001
Scr(μmol/L)	94.026 ± 0.246	94.873 ± 0.337	97.199 ± 1.004	91.531 ± 0.5	92.8 ± 0.501	< 0.001
AST(U/L)	22.497 ± 0.169	21.577 ± 0.225	26.104 ± 0.683	20.006 ± 0.284	24.982 ± 0.403	< 0.001
ALT(U/L)	18.568 ± 0.179	16.451 ± 0.231	23.153 ± 0.659	15.695 ± 0.303	23.564 ± 0.463	< 0.001
GGT(U/L)	25.504 ± 0.482	20.946 ± 0.490	34.906 ± 2.314	23.645 ± 1.113	32.338 ± 1.101	< 0.001
LDH(U/L)	158.053 ± 0.405	151.669 ± 0.573	159.748 ± 1.281	161.443 ± 0.84	168.131 ± 0.893	< 0.001
ALP(U/L)	85.019 ± 0.328	79.677 ± 0.412	86.232 ± 0.947	87.958 ± 0.889	93.468 ± 0.702	< 0.001
TP(g/dl)	7.413 ± 0.005	7.409 ± 0.007	7.473 ± 0.015	7.381 ± 0.011	7.421 ± 0.010	< 0.001
Albumin(g/dL)	4.184 ± 0.004	4.263 ± 0.005	4.22 ± 0.011	4.062 ± 0.008	4.106 ± 0.008	< 0.001
Globulin(g/dL)	2.367 ± 0.016	2.287 ± 0.023	2.51 ± 0.045	2.382 ± 0.037	2.452 ± 0.035	< 0.001
Serum glucose(mmol/L)	5.458 ± 0.020	5.059 ± 0.018	5.545 ± 0.061	5.494 ± 0.045	6.272 ± 0.062	< 0.001
HOMAIR	3.441 ± 0.098	1.977 ± 0.045	3.387 ± 0.351	4.287 ± 0.377	5.929 ± 0.175	< 0.001
eGFR	80.598 ± 0.188	83.752 ± 0.261	82.4 ± 0.543	77.123 ± 0.423	75.908 ± 0.410	< 0.001
FIB-4	0.618 ± 0.005	0.515 ± 0.006	0.718 ± 0.021	0.585 ± 0.011	0.826 ± 0.012	< 0.001
NFS	− 2.312 ± 0.017	− 2.763 ± 0.022	− 2.563 ± 0.045	− 1.877 ± 0.038	− 1.584 ± 0.036	< 0.001
APRI	0.224 ± 0.003	0.215 ± 0.003	0.284 ± 0.016	0.19 ± 0.004	0.245 ± 0.007	< 0.001
Urinary albumin(ug/mL)	38.261 ± 3.237	24.834 ± 3.812	32.935 ± 5.595	46.346 ± 6.593	63.423 ± 10.654	< 0.001
ACR(mg/g)	36.09 ± 3.580	22.824 ± 3.715	34.809 ± 11.299	41.73 ± 5.793	61.031 ± 12.260	< 0.001

(a) Other marital status is widowed, living separately.

Values for categorical variables, as percentage (95% CI); values for continuous variables, as mean ± standard error.

**Figure 2:**
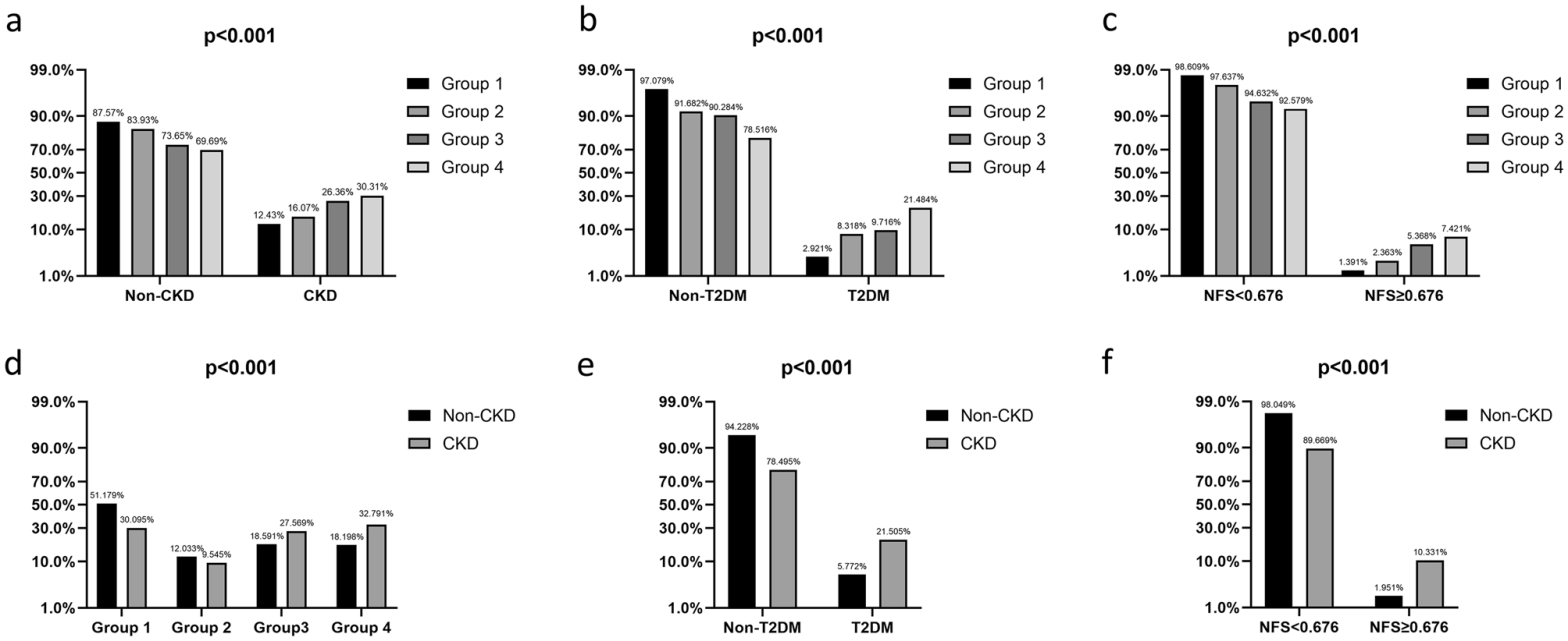
Distribution of participant groups by (**a**) CKD, (**b**) T2DM, (**c**) NFS category and distribution of CKD by (**d**) groups divided by abdominal obesity and MAFLD, (**e**) T2DM, (**f**) NFS category.

In the subgroup analysis, participants with T2DM, advanced fibrosis and CKD were found to have a higher likelihood of being abdominal obese, having MAFLD, or having both abdominal obese and MAFLD (*p* < 0.001). The proportion of participants with T2DM, advanced fibrosis and CKD increased from Group 1 to Group 4 (Fig. 2a,b,c). Correspondingly, participants who had abdominal obesity and MAFLD, T2DM, and advanced liver fibrosis were more likely to have CKD (*p* < 0.001), either(Fig. 2d,e,f).

### Factors associated with CKD

In the univariable and multivariable regression analysis, we found that MAFLD (adjusted OR: 1.392, 95% CI 1.013–1.913, *p* = 0.041), abdominal obesity (adjusted OR 1.456, 95% CI 1.127–1.880, *p* = 0.004), and abdominal obesity with MAFLD (adjusted OR 1.839, 95% CI 1.377–2.456, *p* < 0.001) were all independent risk factors for CKD in fully adjusted model(Table 2). Other factors independently associated with a higher risk of CKD were being female (adjusted OR 1.974, 95% CI 1.575–2.473, *p* < 0.001), being aged 65 or older (adjusted OR 8.735, 95% CI 7.027–10.858, *p* < 0.001), advanced fibrosis(adjusted OR 1.756, 95% CI 1.178–2.619, *p* = 0.006), T2DM (adjusted OR 2.365, 95% CI 1.758–3.183, *p* < 0.001), triglycerides (adjusted OR 1.089, 95% CI 1.010–1.175, *p* = 0.026) and total cholesterol (adjusted OR 1.308, 95% CI 1.205–1.421, *p* < 0.001) (Tab. S1).

**Table 2:** Association of abdominal obesity-MAFLD with risk of CKD.

CKD	Univariable Model		Multivariable Model 1		Multivariable Model 2		Multivariable Model 3	
	OR(95%CI)	*p*	OR(95%CI)	*p*	OR(95%CI)	*p*	OR(95%CI)	*p*
Group 1	1(reference)		1(reference)		1(reference)		1(reference)	
Group 2	1.563(1.178–2.074)	0.002	1.61(1.193–2.174)	0.002	1.548(1.138–2.104)	0.005	1.392(1.013–1.913)	0.041
Group 3	2.758(2.269–3.353)	< 0.001	2.164(1.734–2.701)	< 0.001	2.042(1.636–2.549)	< 0.001	1.456(1.127–1.880)	0.004
Group 4	3.952(3.256–4.795)	< 0.001	3.381(2.718–4.205)	< 0.001	3.140(2.526–3.905)	< 0.001	1.839(1.377–2.456)	< 0.001

Model 1 was adjusted for: age, sex, race.

Model 2 was adjusted for model 1 plus marital status, military service, sedentary behavior.

Model 3 was further adjusted for model 2 plus weight category by BMI, advanced fibrosis by NFS, T2DM, LDL, TG, Tc, albumin.

OR, odds ratio; CI, confidence interval.

### Long‑term mortality in MAFLD and abdominal obese participant

**Figure 3:**
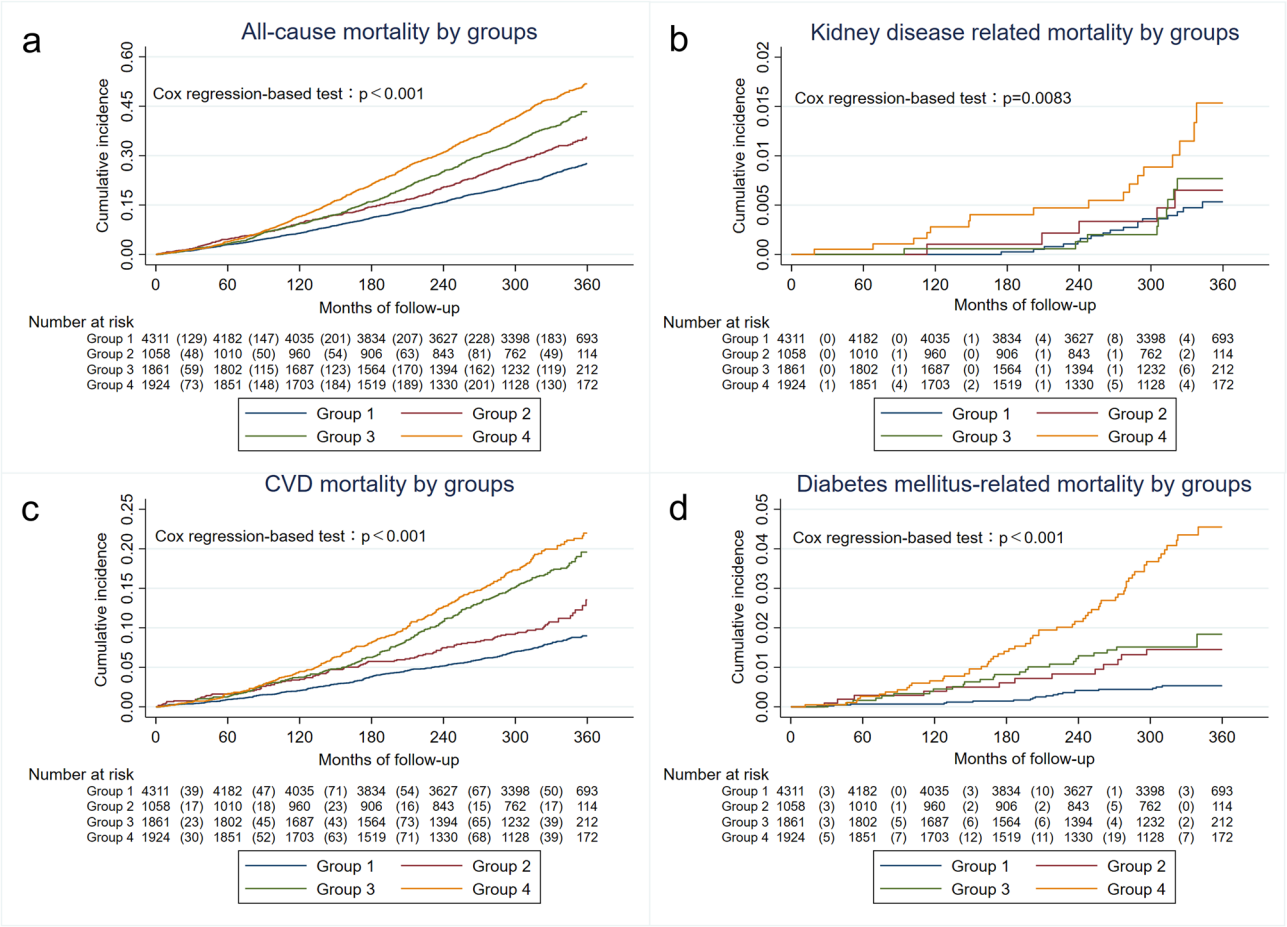
Cumulative mortality among participants in MAFLD-abdominal obesity groups for 30 years. (**a**) all-cause mortality; (**b**) nephritis, nephrotic syndrome and nephrosis related mortality; (**c**) cardiovascular and cerebrovascular related diseases; (**d**) diabetes mellitus related mortality.

We investigated higher 30-years cumulative all-cause mortality among participants among the four groups. We used cox regression analyze and found all-cause mortality of abdominal obese MAFLD group is consistently higher than other groups (*p* < 0.001) (Fig. 3a). Additionally, when categorized by the cause of death, we observed that the cumulative incidence of the abdominal obese MAFLD group was significantly higher than the other groups in kidney disease-related mortality (*p* = 0.0083) (Fig. 3b), cardiovascular and cerebrovascular-related diseases (*p* < 0.001) (Fig. 3c), and diabetes mellitus-related mortality (*p* < 0.001) (Fig. 3d).

### Risk factors of kidney disease-related mortality

As shown in Table 3, abdominal obesity in conjunction with MAFLD was a independent risk factor for kidney-related mortality (adjusted HR: 3.448, CI 1.058–11.239, *p* = 0.040) after adjustment for confounders, while only MAFLD or only abdominal obesity were not. Other risk factors of kidney disease-related mortality were microalbuminuria (adjusted HR: 5.384, 95% CI 1.952–14.845, *p* = 0.001) and total cholesterol (adjusted HR: 1.829, 95% CI 1.555–2.894, *p* = 0.010). In contrast, triglycerides (adjusted HR: 0.545, 95% CI 0.361–0.822, *p* = 0.004), LDL(adjusted HR: 0.796, 95% CI 0.662–0.957, *p* = 0.015), and serum albumin(adjusted HR: 0.141, 95% CI 0.034–0.591, *p* = 0.007) were protective factors of kidney-related mortality (Tab. S2).

**Table 3:** Association of abdominal obesity-MAFLD with risk of kidney disease-related mortality.

kidney disease-related mortality	Univariable model		Multivariable Model 1		Multivariable Model 2		Multivariable model 3	
	HR(95%CI)	*p*	HR(95%CI)	*p*	HR(95%CI)	*p*	HR(95%CI)	*p*
Group 1	1(reference)		1(reference)		1(reference)		1(reference)	
Group 2	1.716(0.400–7.365)	0.468	1.730(0.378–7.922)	0.480	1.599(0.356–7.186)	0.541	1.788(0.391–8.185)	0.454
Group 3	2.358(0.803–6.923)	0.119	2.485(0.837–7.377))	0.101	2.126(0.715–6.321)	0.175	1.348(0.414–4.394)	0.620
Group 4	5.316(2.031–13.913)	0.001	5.617(2.090–15.098)	0.001	4.646(1.698–12.712)	0.003	3.448(1.058–11.239)	0.040

HR, hazard ratio; CI, confidence interval.

Model 1 was adjusted for: age, sex, race.

Model 2 was adjusted for model 1 plus marital status, military service, sedentary behavior.

Model 3 was further adjusted for model 2 plus weight category by BMI, advanced fibrosis by NFS, proteinuria, LDL, TG, Tc, albumin.

### The mediation effect of abdominal obesity in the association between MAFLD and CKD

To further explore the association between MAFLD and CKD, we conducted covariate-adjusted causal mediation analyses. As shown in Table 4, we observed a significant indirect mediation effect of MAFLD on CKD through several obesity measurement indexes, Notably, indicators that represent abdominal obesity count higher proportion of mediation effect than traditional BMI(32.63%), such as WC(65.23%), WHR(70.68%), WHtR(68.21%) and BRI(62.68%). Moreover, LAP, which represents lipid accumulation, accounts for the highest proportion mediated, amounting to 71.98% of the total effect.

**Table 4:** Mediation analysis for the associations between MAFLD and CKD.

Independent variable	Mediator	Direct effect	Indirect effect	Proportion of mediation (%)
		Coefficient (95% CI)	Coefficient (95% CI)	
MAFLD	BMI	0.02001 (0.00623, 0.03340)	0.00969 (0.00524, 0.01426)	32.63
	WC	0.01033 (− 0.00547, 0.02504)	0.01937 (0.01438, 0.02467)	65.23
	WHR	0.00871 (− 0.00620, 002,271)	0.02099 (0.01623, 0.02554)	70.68
	WHtR	0.00944 (− 0.00438, 0.02388)	0.02026 (0.01520, 0.02519)	68.21
	ABSI	0.02019 (0.00735, 0.03417)	0.00951 (0.00691, 0.01219)	32.03
	BRI	0.01108 (− 0.00438,0.02608)	0.01862(0.01328, 0.02423)	62.68
	VAI	0.01775 (− 0.00493,0.03245)	0.01196(0.00785, 0.02885)	40.25
	LAP	0.00832 (− 0.00681, 0.02137)	0.02138 (0.01494, 0.02085)	71.98

Number of bootstrap samples for percentile bootstrap confidence intervals: 1000.

Adjusted for sex, age, race, PIR, marital status, military service and sedentary behavior.

Proportion mediated = indirect effect/ (direct effect + indirect effect).

## Discussion

As ongoing research continues to explore the utilization of MAFLD as a more comprehensive and refined term and definition for characterizing what appears to be a metabolically based fatty liver disease, our study aimed to examine the demographic and clinical characteristics, mortality, and the complex association among MAFLD, abdominal obesity and CKD. In our study, we found that 32.584% of individuals had MAFLD, and 41.371% were abdominal obese. Among the abdominal obese participants, 50.844% had MAFLD, which was significantly higher than the 19.698% observed in non-abdominal obese participants. Among those with MAFLD, abdominal obese MAFLD individuals were more likely to be female, older than 65 years, and exhibit higher prevalence of T2DM, advanced liver fibrosis and CKD. Additionally, we observed a higher distribution of advanced liver fibrosis, T2DM, abdominal obesity, and MAFLD in the CKD population. These findings were highlighted in our multivariate logistic analysis where we identified several independent risk factors for CKD, including MAFLD, abdominal obesity, T2DM, being female, age over 65, and sedentary behavior, advanced fibrosis, triglycerides and total cholesterol.

In our study, we found participants with abdominal obesity and MAFLD were more likely to be female. Similar findings have been reported in other literatures. In the research conducted by Dao et al., there was a higher prevalence of obesity MAFLD in females than males (62.6% vs. 47.6%; *p* < 0.001)^25^. Previous research has demonstrated that there were significantly more females than males with MAFLD in age subgroups older than 40 years and there was a sharp rise in the prevalence of MAFLD in perimenopausal and postmenopausal women^26,27^, a period during which the decrease in oestrogen levels can lead to fat redistribution and lead to metabolic disorders, including MAFLD^28^. Furthermore, abdominal obesity in women is a well-documented risk factor for polycystic ovary syndrome(PCOS) while a number of studies have suggested the close correlation between PCOS and NAFLD^29^. A meta-analyze involving 7148 participants has reported that premenopausal PCOS patients are associated with 2.5-fold increase in the risk of NAFLD^30^. In conclusion, it is reasonable to suggest that the high proportion of women with abdominal obesity and MAFLD may be associated with decreased oestrogen levels and co-morbid of PCOS.

We also examined the mortality and found that over a follow-up time of 30 years, the all cause mortality among participants with abdominal obesity and MAFLD was always higher compared to the other groups. Combined with the results of previous baseline data and correlation analysis, we further followed up mortality related to T2DM, cardiovascular and cerebrovascular disease, and kidney disease, and found that participants with both abdominal obesity and MAFLD had the highest mortality among these three cause of mortality. Therefore, we took one additional step to perform a cox regression analysis and confirmed that abdominal obesity plus MAFLD was indeed a risk factor for kidney-related mortalityafter fully adjusted for known prognostic factors. However, only abdominal obesity, as well as MAFLD alone, were not found to be independent risk factors for kidney disease-related death in our study. This finding suggests that the increased mortality associated with kidney disease may be attributed to possible combined effect of abdominal obesity and MAFLD. This relationship has not been demonstrated in previous studies, thus motivated us to conduct causal mediation effect analysis to investigate the potential role of abdominal obesity as a mediator in the association between MAFLD and CKD.

In causal mediation analysis, we found that obesity mediate the relationship between MAFLD and CKD. Notably, when compared to BMI, obesity measurement indexes that better represent abdominal obesity or accumulation of lipid, such as WC, WHR, WHtR, BRI and LAP^12,21,31,32^, exhibit higher proportion of mediation. This intriguing observation provides a novel perspective on the potential role of visceral fat or accumulated lipid in influencing the association between MAFLD and CKD. It is important to recognize that fat deposition is not limited to adipose tissue but can also occur in non-adipose tissues such as the liver and kidneys and consequently impacts organ function, which is closely associated with both MAFLD and CKD. In both conditions, excessive lipid accumulation exacerbates inflammation, oxidative stress, and organ structural damage by lipotoxicity^33–35^. Hence, it can be argued that the use of WC as a diagnostic criterion for metabolic disorders in MAFLD proves to be a superior measure of obesity compared to BMI, which is the diagnostic basis for obesity in MAFLD, in exploring the association of MAFLD with patients with CKD.

Abdominal obesity may also contribute to the cross-linking of MAFLD and CKD by increasing the risk of insulin resistance and diabetes mellitus^36,37^. In our multivariate analysis, we found that T2DM, abdominal obesity, and MAFLD are all independent risk factors of CKD. There are several studies that supported our results to certain extent and found the influential role of abdominal obesity/adiposity in diabetic kidney disease or CKD caused by diabetes mellitus^38–41^. It is noteworthy that both abdominal obesity and diabetes mellitus are included in the diagnostic criteria for MAFLD, and their association with MAFLD has been demonstrated in many studies^42^. A cross-sectional study using of 12,571 individuals data from NHANES III has found that patients with MAFLD with coexisting T2DM had a higher prevalence of CKD than their counterparts without diabetes(46.99% vs. 24.22%)^43^. Another longitudinal study has shown that the clustering of obesity, visceral obesity, and fatty liver disease markedly increased the risk of T2DM in men (adjusted HR 10.5, 95% CI 8.0–13.8) and women (adjusted HR 30.0, 95% CI 18.0–50.0)^44^. What’s more, the patatin-like phospholipase domain-containing 3 (PNPLA3) rs738409 gene is considered the strongest genetic determinant of fatty liver disease, is also highly expressed on renal podocytes and contributes to renal dysfunction^35,45,46^. Several studies have found that this genetic variant is primarily associated with insulin resistance or T2DM patients with obesity^47^. Consequently, extrapolating based on our results and previous studies, abdominal obesity may further enhance the connection between MAFLD and CKD partly through the presence of T2DM.

Our study revealed that liver fibrosis is more prevalent and severe in individuals with abdominal obese MAFLD compared to non-abdominal individuals with MAFLD, as well as in those with CKD compared to those without CKD. Our results are consistent with a community-based prospective study with an average follow-up duration of 4.4 years indicated that the development of NAFLD and progression of fibrosis are linked to an elevated risk of incident CKD^48^. In addition, NAFLD may exacerbate systemic and hepatic insulin resistance, cause atherogenic dyslipidemia and adipose accumulation, and release a variety of pro-inflammatory, pro-coagulant, pro-oxidant, and pro-fibrogenic mediators that may contributes directly to endothelial dysfunction and tubulointerstitial fibrosis and result in the development and progression of CKD^49,50^. It has been observed that visceral obesity, rather than elevated BMI, has a stronger correlation with the degree of fibrosis in patients with chronic hepatitis C^51^, suggesting a potential connection between abdominal obesity and hepatic fibrosis. Several studies have shown that the PNPLA3 I148M variant was also associated with an increased risk of steatosis and fibrosis in liver and kidney, and fat accumulation in this process is causally linked with liver fibrosis and kidney disease progression^35,52,53^. Thus, our findings highlight the potential role of liver fibrosis relationship between abdominal obesity, MAFLD and CKD. However, understanding the mechanisms and genetic factors that contribute to the onset and progression of these diseases necessitates further investigation through basic medical and prospective studies.

There still existed some limitations in this study. Firstly, NHANES III did not include an over-sampling of Asian Americans and participants with race and ethnicities other than white, black, and Hispanic, so our data may not be generalizable to the Asian American and other race and ethnicity groups. Second, until now, liver biopsy has been the gold standard for dentification of steatosis and advanced hepatic fibrosis, but due to limitations in the NHANES study, we were only able to assess this using noninvasive tests with as much specificity and sensitivity as possible. Third, our study did not include alcohol consumption, smoking, and other possible confounders in the analysis, given that the high level of missing data in this section would have significantly reduced the sample size we included, but these factors may have some influence on the progression of the development of abdominal obesity, MAFLD, and CKD. Moreover, according to the KDIGO Clinical Practice Guidelines, a diagnosis of CKD requires a decline in eGFR and/or presence of kidney damage (such as proteinuria) persisting a minimum of three months. However, due to the limitations of the NHANES III database, the diagnosis of CKD in this article is limited to the criteria of eGFR < 60 mL/min/1.73 m2 and/or ACR ≥ 30 mg/g, without the criterion of duration of not less than 3 months.

## Conclusions

Using the data from NHANES III from 1988 to 1994 and the mortality follow-up survey in 2019, we found participants with abdominal obesity and MAFLD were more likely to be female, older and have higher prevalence of T2DM, advanced liver fibrosis and CKD. The abdominal obesity in conjunction with MAFLD had about 3.5-fold increase in the risk of kidney disease-related mortality and the highest cumulative mortality in the 30 years follow-up period. Abdominal obesity may mediate the association between MAFLD and CKD. WC, WHR, LAP are superior indicators of the mediation effect of MAFLD on CKD compared to BMI. Consequently, it is crucial to identify and select appropriate indicators to assess abdominal obesity. Individuals with either MAFLD or abdominal obesity should undergo screening CKD at an early stage, allowing for the implementation of effective interventions to reduce the associated risk of mortality.

### Supplementary Information

Supplementary Tables.

## Data Availability

The datasets used in the current study are publicly available at https://wwwn.cdc.gov/nchs/nhanes.
