# Supplementary material for: Associations between metabolic dysfunction-associated fatty liver disease, chronic kidney disease, and abdominal obesity: a national retrospective cohort study
Source: Sci Rep. 2024 Jun 2;14:12645. doi: 10.1038/s41598-024-63386-0 (PMC11144701; doi:10.1038/s41598-024-63386-0)
Supplement: Supplementary file 1 — Supplementary Tables. [file 41598_2024_63386_MOESM1_ESM.docx]

| Table S1. Other factors associated with CKD | | | | | | | | | | | |
| --- | --- | --- | --- | --- | --- | --- | --- | --- | --- | --- | --- |
| CKD | Univariable model | |  | Multivariable model 1 | |  | Multivariable model 2 | |  | Multivariable model 3 | |
|  | OR(95%CI) | p |  | OR(95%CI) | p |  | OR(95%CI) | p |  | OR(95%CI) | p |
| Gender |  |  |  |  |  |  |  |  |  |  |  |
| Male | 1(reference) |  |  | 1(reference) |  |  | 1(reference) |  |  | 1(reference) |  |
| Female | 1.739(1.490-2.029) | <0.001 |  | 1.691(1.410-2.028) | <0.001 |  | 1.874(1.516-2.317) | <0.001 |  | 1.974(1.575-2.473) | <0.001 |
| Race |  |  |  |  |  |  |  |  |  |  |  |
| Non-Hispanic White | 1(reference) |  |  | 1(reference) |  |  | 1(reference) |  |  | 1(reference) |  |
| Non-Hispanic Black | 0.891(0.771-1.030) | 0.118 |  | 0.948(0.803-1.118) | 0.524 |  | 0.999(0.836-1.193) | 0.988 |  | 0.910(0.748-1.107) | 0.347 |
| Mexican American | 0.524(0.446-0.616) | <0.001 |  | 0.587(0.488-0.705) | <0.001 |  | 0.594(0.491-0.718) | <0.001 |  | 0.571(0.468-0.696) | <0.001 |
| Other race | 0.808(0.578-1.130) | 0.212 |  | 1.011(0.699-1.461) | 0.955 |  | 1.055(0.721-1.543) | 0.785 |  | 0.991(0.670-1.466) | 0.963 |
| Age |  |  |  |  |  |  |  |  |  |  |  |
| ＜65 | 1(reference) |  |  | 1(reference) |  |  | 1(reference) |  |  | 1(reference) |  |
| ≥65 | 12.142(10.123-14.564) | <0.001 |  | 11.097(9.128-13.491) | <0.001 |  | 10.306(8.401-12.644) | <0.001 |  | 8.735(7.027-10.858) | <0.001 |
| Marriage status | |  |  |  |  |  |  |  |  |  |  |
| Legally married | 1(reference) |  |  |  |  |  | 1(reference) |  |  | 1(reference) |  |
| Divorced/separated | 0.962(0.748-1.237) | 0.762 |  |  |  |  | 0.931(0.709-1.223) | 0.608 |  | 0.938(0.709-1.241) | 0.654 |
| Never married | 0.429(0.328-0.561) | <0.001 |  |  |  |  | 0.661(0.494-0.884) | 0.005 |  | 0.754(0.557-1.021) | 0.068 |
| Other(a) | 1.702(1.355-2.137) | <0.001 |  |  |  |  | 0.933(0.707-1.232) | 0.625 |  | 0.948(0.711-1.263) | 0.713 |
| Military service |  |  |  |  |  |  |  |  |  |  |  |
| No | 1(reference) |  |  |  |  |  | 1(reference) |  |  | 1(reference) |  |
| Yes | 0.699(0.580-0.843) | <0.001 |  |  |  |  | 0.748(0.574-0.976) | 0.032 |  | 0.782(0.598-1.024) | 0.073 |
| Sedentary behavior | |  |  |  |  |  |  |  |  |  |  |
| No | 1(reference) |  |  |  |  |  | 1(reference) |  |  | 1(reference) |  |
| Yes | 1.34(1.131-1.587) | 0.001 |  |  |  |  | 1.148(0.942-1.399) | 0.172 |  | 1.129(0.921-1.383) | 0.242 |
| Advanced fibrosis by NFS | |  |  |  |  |  |  |  |  |  |  |
| No | 1(reference) |  |  |  |  |  |  |  |  | 1(reference) |  |
| Yes | 6.350(4.551-8.858) | <0.001 |  |  |  |  |  |  |  | 1.756(1.178-2.619) | 0.006 |
| T2DM |  |  |  |  |  |  |  |  |  |  |  |
| No | 1(reference) |  |  |  |  |  |  |  |  | 1(reference) |  |
| Yes | 4.573(3.582-5.839) | <0.001 |  |  |  |  |  |  |  | 2.365(1.758-3.183) | <0.001 |
| Weight category by BMI | |  |  |  |  |  |  |  |  |  |  |
| Non-obese | 1(reference) |  |  |  |  |  |  |  |  | 1(reference) |  |
| Obese | 2.202(1.864-2.600) | <0.001 |  |  |  |  |  |  |  | 1.249(0.973-1.603) | 0.081 |
| TG(mmol/L) | 1.314(1.227-1.408) | <0.001 |  |  |  |  |  |  |  | 1.089(1.010-1.175) | 0.026 |
| LDL(mmol/L) | 1.036(0.991-1.083) | 0.120 |  |  |  |  |  |  |  | 0.995(0.949-1.045) | 0.852 |
| Tc(mmol/L) | 1.636(1.533-1.745) | <0.001 |  |  |  |  |  |  |  | 1.308(1.205-1.421) | <0.001 |
| Albumin(g/dL) | 0.384(0.311-0.474) | <0.001 |  |  |  |  |  |  |  | 0.842(0.644-1.101) | 0.209 |
| Model 1 was adjusted for: age, sex, race  Model 2 was adjusted for model 1 plus marriage status, military service, sedentary behavior  Model 3 was further adjusted for model 2 plus weight category by BMI, advanced fibrosis by NFS, T2DM, LDL, TG, Tc, albumin  OR, odds ratio; CI, confidence interval | | | | | | | | | | | |

| Table S2. Other risk factors of kidney disease-related mortality | | | | | | | | | | | |
| --- | --- | --- | --- | --- | --- | --- | --- | --- | --- | --- | --- |
| kidney disease-related mortality | Univariable model | |  | Multivariable model 1 | |  | Multivariable model 2 | |  | Multivariable model 3 | |
|  | HR(95%CI) | p |  | HR(95%CI) | p |  | HR(95%CI) | p |  | HR(95%CI) | p |
| Gender |  |  |  |  |  |  |  |  |  |  |  |
| Male | 1(reference) |  |  | 1(reference) |  |  | 1(reference) |  |  | 1(reference) |  |
| Female | 0.901(0.405-2.005) | 0.799 |  | 0.765(0.320-1.833) | 0.549 |  | 0.894(0.325-2.457) | 0.828 |  | 0.481(0.148-1.564) | 0.224 |
| Race |  |  |  |  |  |  |  |  |  |  |  |
| Non-Hispanic White | 1(reference) |  |  | 1(reference) |  |  | 1(reference) |  |  | 1(reference) |  |
| Non-Hispanic Black | 1.088(0.525-2.255) | 0.820 |  | 1.052(0.505-2.192) | 0.892 |  | 1.304(0.638-2.665) | 0.467 |  | 0.698(0.286-1.702) | 0.429 |
| Mexican American | 0.488(0.212-1.126) | 0.093 |  | 0.417(0.176-0.985) | 0.046 |  | 0.429(0.173-1.064) | 0.068 |  | 0.427(0.164-1.111) | 0.081 |
| Other race | 0.531(0.071-3.969) | 0.537 |  | 0.507(0.066-3.877) | 0.513 |  | 0.585(0.073-4.671) | 0.613 |  | 0.432(0.050-3.726) | 0.445 |
| Marriage status | |  |  |  |  |  |  |  |  |  |  |
| Legally married | 1(reference) |  |  |  |  |  | 1(reference) |  |  | 1(reference) |  |
| Divorced/separated | 0.433(0.161-1.166) | 0.098 |  |  |  |  | 0.422(0.16-1.114) | 0.082 |  | 0.349(0.123-0.99) | 0.048 |
| Never married | 0.155(0.031-0.760) | 0.022 |  |  |  |  | 0.207(0.041-1.046) | 0.057 |  | 0.222(0.043-1.156) | 0.074 |
| Other(a) | 1.361(0.446-4.150) | 0.588 |  |  |  |  | 1.383(0.445-4.303) | 0.575 |  | 1.411(0.44-4.524) | 0.563 |
| Military |  |  |  |  |  |  |  |  |  |  |  |
| No | 1(reference) |  |  |  |  |  | 1(reference) |  |  | 1(reference) |  |
| Yes | 0.515(0.210-1.260) | 0.146 |  |  |  |  | 0.652(0.207-2.051) | 0.464 |  | 0.755(0.220-2.592) | 0.655 |
| Sedentary behavior | |  |  |  |  |  |  |  |  |  |  |
| No | 1(reference) |  |  |  |  |  | 1(reference) |  |  | 1(reference) |  |
| Yes | 1.260(0.518-3.064) | 0.610 |  |  |  |  | 1.203(0.477-3.036) | 0.696 |  | 1.174(0.447-3.082) | 0.745 |
| Advanced fibrosis by NFS | |  |  |  |  |  |  |  |  |  |  |
| No | 1(reference) |  |  |  |  |  |  |  |  | 1(reference) |  |
| Yes | 3.213(0.780-13.232) | 0.106 |  |  |  |  |  |  |  | 1.250(0.249-6.264) | 0.786 |
| Weight category by BMI | |  |  |  |  |  |  |  |  |  |  |
| No | 1(reference) |  |  |  |  |  |  |  |  | 1(reference) |  |
| Yes | 3.029(1.315-6.977) | 0.009 |  |  |  |  |  |  |  | 1.283(0.475-3.465) | 0.622 |
| Proteinuria | |  |  |  |  |  |  |  |  |  |  |
| Normal range | 1(reference) |  |  |  |  |  |  |  |  | 1(reference) |  |
| Microalbuminuria | 6.345(2.489-16.173) | <0.001 |  |  |  |  |  |  |  | 5.384(1.952-14.845) | 0.001 |
| Macroalbuminuria | 7.974(1.624-39.145) | 0.011 |  |  |  |  |  |  |  | 2.345(0.457-12.019) | 0.307 |
| TG(mmol/L) | 1.059(0.968-1.158) | 0.212 |  |  |  |  |  |  |  | 0.545(0.361-0.822) | 0.004 |
| LDL(mmol/L) | 0.870(0.692-1.095) | 0.235 |  |  |  |  |  |  |  | 0.796(0.662-0.957) | 0.015 |
| Tc(mmol/L) | 1.695(1.230-2.335) | 0.001 |  |  |  |  |  |  |  | 1.829(1.155-2.894) | 0.010 |
| Albumin(g/dL) | 0.199(0.072-0.549) | 0.002 |  |  |  |  |  |  |  | 0.141(0.034-0.591) | 0.007 |
| Model 1 was adjusted for: age, sex, race  Model 2 was adjusted for model 1 plus marriage status, military service, sedentary behavior  Model 3 was further adjusted for model 2 plus weight category by BMI, advanced fibrosis by NFS, proteinuria, LDL, TG, Tc, albumin  HR, hazard ratio; CI, confidence interval | | | | | | | | | | | |
